# Global trends in machine learning applied to clinical research in liver cancer: Bibliometric and visualization analysis (2001–2024)

**DOI:** 10.1097/MD.0000000000040790

**Published:** 2024-12-06

**Authors:** Enba Zhuo, Wenzhi Yang, Yafen Wang, Yanchao Tang, Wanrong Wang, Lingyan Zhou, Yanjun Chen, Pengman Li, Bangjie Chen, Weimin Gao, Wang Liu

**Affiliations:** a Department of Anesthesiology, The First Affiliated Hospital of Anhui Medical University, Hefei, China; b First Clinical College, Anhui Medical University, Hefei, China; c Department of Radiation Oncology, The First Affiliated Hospital of Anhui Medical University, Hefei, China; d Department of Oncology, The First Affiliated Hospital of Anhui Medical University, Hefei, China; e Department of General Surgery, Sanya Central Hospital (The Third People’s Hospital of Hainan Province), Sanya, China.

**Keywords:** bibliometric analysis, clinical diagnosis, liver cancer (LC), machine learning (ML), treatment strategies

## Abstract

This study explores the intersection of liver cancer and machine learning through bibliometric analysis. The aim is to identify highly cited papers in the field and examine the current research landscape, highlighting emerging trends and key areas of focus in liver cancer and machine learning. By analyzing citation patterns, this study sheds light on the evolving role of machine learning in liver cancer research and its potential for future advancements.

## 1. Introduction

Liver cancer (LC) is one of the leading causes of cancer-related deaths worldwide.^[1,2]^ Ranking as the fourth most common cause of cancer mortality globally,^[3]^ Unlike other major cancers, LC continues to rise in incidence annually.^[2]^ Hepatocellular carcinoma (HCC), the most common form of primary liver cancer, poses a significant risk, particularly to patients with cirrhosis.^[4–6]^ By 2025, an estimated 1 million new cases of LC are projected, with HCC accounting for over 90% of them.^[7]^ Based on current trends, HCC is expected to become the third leading cause of cancer-related deaths by 2030.^[7–9]^ Highlighting its growing public health burden.

Although significant progress has been made in understanding LC’s mechanisms, diagnosis, and treatment,^[7,10]^ many patients are diagnosed at advanced stages due to the aggressive nature of the disease and its tendency for metastasis.^[11,12]^ Surgical resection remains the most effective treatment, but only a small fraction of patients (5–15%) are eligible for surgery due to the advanced stage at diagnosis^[2,13]^; This late detection severely limits the ability to gather information on early disease progression, contributing to poor prognosis. Therefore, early diagnosis, accurate prognosis, personalized treatment, and ongoing monitoring are critical for improving outcomes in LC.

In recent years, machine learning, a core component of artificial intelligence, has shown great promise in healthcare, particularly in the diagnosis, treatment, and prognosis of LC. ML uses algorithms to analyze large datasets, detect patterns, and improve decision-making. Techniques such as decision trees, support vector machines, random forests, and deep learning have been applied to various aspects of LC research.^[14]^ For example, ML has been used to develop prediction models based on LC metabolic genes, helping to improve risk stratification, prognosis, and personalized treatment approaches for patients.^[15]^ ML combined with contrast-enhanced computed tomography has also been applied to predict tumor recurrence after surgery and to guide therapy for patients with recurrent LC.^[16,17]^ Additionally, prediction models have been used to optimize treatment selection^[18]^ and facilitate telemedicine for patients with complex cases.^[19]^ While ML has the potential to transform LC diagnosis and treatment, there is a lack of comprehensive and systematic analyses of this field from a quantitative perspective. Existing meta-analyses have been criticized for their limited scope and lack of convincing results,^[20,21]^ often influenced by the subjective interpretations of researchers.^[22]^ Many studies have relied on narrow literature reviews or clinical experiences, offering limited insights into the broader trends and challenges in LC research.^[23]^ Due to the dynamic nature of LC, it is essential that practitioners be abreast of new developments and significant shifts in the discipline as a whole.^[24]^ Bibliometric analysis, a method that quantitatively examines research output and trends, can address some of these limitations. Tools such as CiteSpace and VOSviewer are used to map knowledge and track the evolution of research fields.^[22]^ These visual analytics, combined with traditional reviews, provide valuable insights into the development of disease treatments and clinical guidelines, particularly for rapidly evolving fields like LC.^[24]^

Given the growing interest in ML applications for LC research, a thorough examination of the current state and future trajectory of this field is essential. However, no comprehensive study has yet measured the scientific contributions of ML in LC. This paper aims to fill that gap by using bibliometric analysis to map the development of ML in LC research from 2001 to 2024. Bibliometric analysis can be used to analyze research trends and hot spots and provide new ideas and theoretical references for future research on the diagnosis and treatment of ML-driven LC.^[25,26]^

## 2. Materials and methods

### 2.1. Data source and search strategy

**Figure 1.**
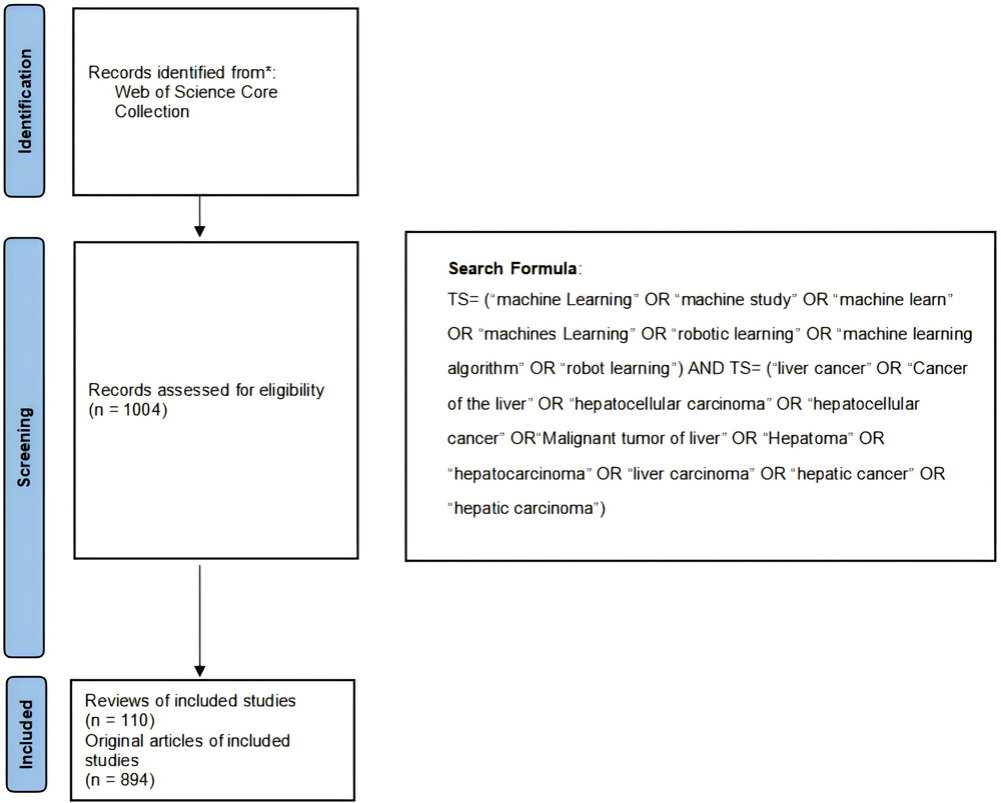
Flow diagram of screening process related to LC/ML. LC/ML = liver cancer and machine learning.

Web of Science database features prestigious publications from all around the globe in the fields of natural science, social science, the arts, and many more. Top mainstream academic journals in the natural sciences are included in the Science Core Collection (WoSCC). Hence, we opted for the WoSCC as our primary search source. To mitigate any potential bias stemming from database updates, all searches were performed on a single day, specifically on August 15, 2024. The search strategy employed the use of the subject term “advanced search” method, with the search terms TS = “machine learning” and “liver cancer” and their corresponding synonyms. In order to conduct a thorough search for pertinent literature, terms associated with machine learning and liver cancer were extracted from PubMed’s Medical Subject Headings. The selection criteria were as follows (full search approach presented in Supplementary Material S1, Supplemental Digital Content, http://links.lww.com/MD/O114): Our search parameters were as follows: a date range of January 1, 2001, through August 15, 2024; a restriction to just “article” and “review” literature; and an English language option. Thirdly, the language selection was limited to English. The final number of articles retrieved after filtering was 1004, with 894 being “Articles” and 110 being “Reviews” (Fig. 1). Data mining and analysis were carried out by 3 researchers (Enba Zhuo, Yafen Wang, and Bangjie Chen). We exported the records to plain text files after extracting all relevant data, including titles, authors, institutions, countries (regions), publication years, and keywords, from the raw data.

### 2.2. Bibliometric analysis

We exported publications that meet the inclusion criteria in plain text format with “full record and cited references” and named the file “download_xxx.txt” and import this file into VOSviewer 1.6.20 and CiteSpace 6.3.R1 for scientific knowledge graph visualization. The following are the parameter settings for the 2 software. In VOSviewer, the standardized method was set to association strength, and the minimum thresholds for countries, institutions, and authors publications were set to 5, 10, and 5, and the minimum thresholds for cited journals and references frequency were set to 266 and 34, respectively. The layout of the references was adjusted by using Pajek64 5.19. The minimum frequency of keyword occurrence was set to 20. In CiteSpace, we set the time span from January 2001 to August 2024, and the year of each slice was 1. We selected “keyword” and “reference” as the node types to draw the double-graph overlay of journals, reference outburst, and keyword outburst. These maps of scientific knowledge visually show trends and directions in the field of liver cancer and machine learning.^[27,28]^

## 3. Results

### 3.1. Characterization of the overall distribution

#### 3.1.1. Annual trends in publications

**Figure 2.**
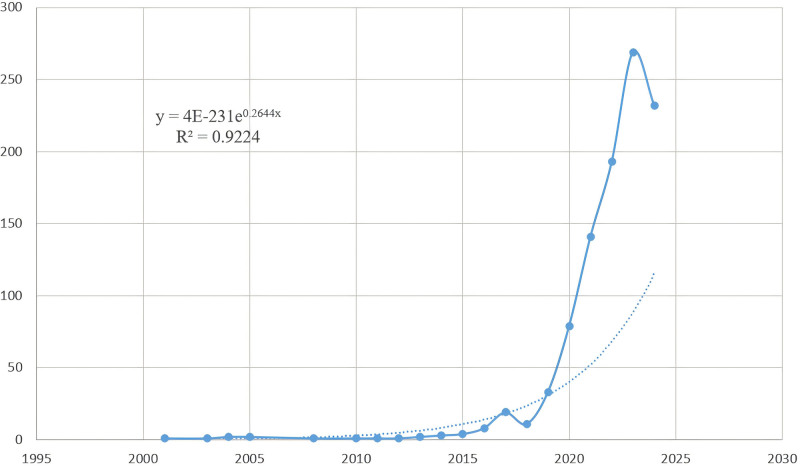
The annual number of published LC/ML studies, 2004 to 2024. LC/ML = liver cancer and machine learning.

Following a thorough manual patient screening process, it has been determined that the cumulative quantity of literature pertaining to liver cancer and machine learning (LC/ML) from the years 2001 to 2024 amounts to 1004. The examination of the temporal progression of published articles might provide insights into the research patterns, as seen in Figure 2. From 2001 to 2012, the research area under consideration has not received significant attention, as evidenced by the relatively small number of papers (Np) published each year, with Np below 10 (n = 10, 1.00%). The growth rate of publications remained slow from 2013 to 2015 (n = 9, 0.90%). However, there was a remarkable exponential increase in the number of papers from 2016 to 2024 (n = 985, 98.11%), reaching its peak at 269 papers in 2023. It is worth noting that there was a slight decrease in 2018, which could be attributed to factors such as the limited innovativeness of the papers published that year and the relatively lower proficiency in manuscript writing. Furthermore, we developed a polynomial regression model (*f* (x)  = *p*_0_*x*^n^ + *p*_1_*x*^n−1^ + *p*_2_x^n−2^ + *p*_3_*x*^n−3^+…+*p*_n_) using Microsoft Office Excel 2019 in order to forecast the quantity of Np publications expected to be released in the year 2025. A model for the temporal prediction curve was constructed by fitting the available data using the equation $y=4E-231e^{0.2644x}$. A statistically significant correlation was established between the number of publications and the year (*R*²=0.9224), indicating a strong link. The curve and the data exhibited a good match. The exponential expansion of Np in the domains associated with LC/ML in recent times indicates a notable surge in scholarly interest in investigating this particular topic. In conclusion, it is our contention that the future outlook for machine learning research in the subject of natural language processing is quite promising.

#### 3.1.2. Analysis of citations

Table 1 presents the top 10 most cited global papers in LC/ML. Leading the list is Predicting Hepatitis B Virus-Positive Metastatic Hepatocellular Carcinomas Using Gene Expression Profiling and Supervised Machine Learning by Qing-hai Ye et al, with 706 citations. This landmark paper is widely cited because it was the first to use supervised machine learning algorithms to identify molecular markers that classify patients with metastatic hepatocellular carcinoma and pinpoint genes linked to metastasis and patient survival.^[29]^ Ranking second, with 322 citations, is Long non-coding RNAs and complex diseases: from experimental results to computational models by Xing Chen et al. This article introduces an advanced computational model to identify disease-associated lncRNAs on a large scale and validates the findings experimentally.^[30]^ Third on the list, with 221 citations, is Aberrant Lipid Metabolism in Hepatocellular Carcinoma Revealed by Plasma Metabolomics and Lipid Profiling by Andrew D. Patterson, which explored lipid metabolism disruptions in liver cancer. Notably, 4 of the top 10 cited papers were published in 2017, potentially indicating a rise in the quality and impact of research in the LC/ML field during this period.

**Table 1:** The top 10 global cited papers based on total citations in LC/ML.

Paper	DOI	Total citations	TC per year	Normalized TC
Ye QH, 2003, Nat Med	10.1038/nm843	706	32.09	1
Chen X, 2017, Brief Bioinform	10.1093/bib/bbw060	491	61.38	5.59
Hao XK, 2017, P Natl Acad Sci USA	10.1073/pnas.1703577114	322	40.25	3.67
Patterson AD, 2011, Cancer Res	10.1158/0008-5472.CAN-11-0885	221	15.79	1
Singal AG, 2013, Am J Gastroenterol	10.1038/ajg.2013.332	183	15.25	1.96
Chiappini F, 2017, Sci Rep-UK	10.1038/srep46658	152	19	1.73
Papanikolaou N, 2020, Cancer Imaging	10.1186/s40644-020-00311-4	148	29.6	5.13
Anafi RC, 2017, Proc natl Acad Sci USA	10.1073/pnas.1619320114	143	17.88	1.63
Feng ST, 2019, Eur Radiol	10.1007/s00330-018-5935-8	134	22.33	3.65
Ma LC, 2021, J Hepatol	10.1016/j.jhep.2021.06.028	126	31.5	8.13

LC/ML = liver cancer and machine learning, TC = total citations.

### 3.2. Analysis of journal distribution

**Figure 3.**
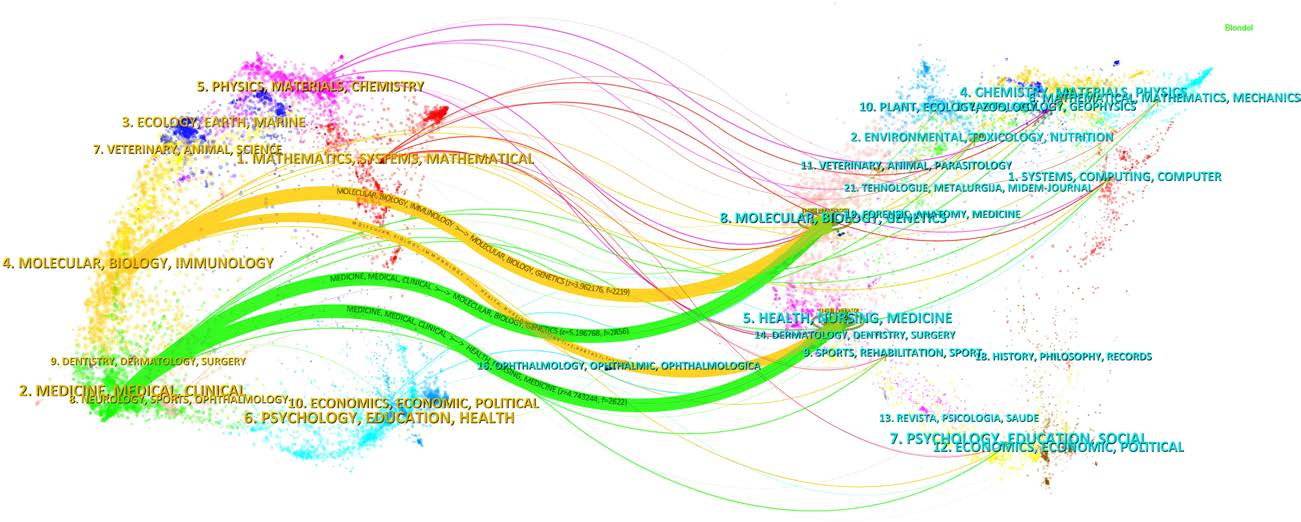
The dual-map overlay of LC/ML research-related journals. LC/ML = liver cancer and machine learning.

The publications analyzed were distributed across 423 scholarly journals. Table 2 ranks the top ten journals based on total citations (TC) and H-Index. Nature Medicine had the highest TC at 706, followed by European Radiology (TC = 565) and Scientific Reports (TC = 524). In terms of H-Index, Frontiers in Oncology ranked first with a score of 13, while Scientific Reports came second with 12. Cancers and the World Journal of Gastroenterology both shared third place with an H-Index of 9. Figure 3 provides a spatial representation of the study’s distribution across prominent research fields. The map is divided into 2 sections: the left side displays the citation map, while the right side shows the cited map. Each point on the map represents a journal, with publications in medicine and clinical research (shown in green) and molecular biology and immunology (shown in orange) demonstrating a wide dispersion across these fields.

**Table 2:** Distribution of top 10 local impact journals in LC/ML.

Rank	Journals	TC	Journals	H-Index
1	Nature Medicine	706	Frontiers in Oncology	13
2	European Radiology	565	Scientific Reports	12
3	Scientific Reports	524	Cancers	9
4	Briefings in Bioinformatics	509	World Journal of Gastroenterology	9
5	Frontiers in Oncology	500	European Radiology	8
6	Proceedings of the National Academy of Sciences of the United States of America	481	Computers in Biology and Medicine	7
7	Journal of Hepatology	383	Diagnostics	7
8	Computers in Biology and Medicine	231	Journal of Hepatology	7
9	Journal of Biomedical Informatics	221	Frontiers in Genetics	6
10	Cancer Research	221	Frontiers in Immunology	6

LC/ML = liver cancer and machine learning, TC = total citations.

### 3.3. Analysis of relevant yields

The dataset includes over 6965 authors and 4497 institutions. To minimize duplications due to abbreviations, we used complete author names alongside their respective Web of Science Researcher IDs to extract and evaluate the number of publications. Table 3 lists the top 10 countries (regions) by Np, with the People’s Republic of China leading with 537 publications, followed by the United States with 210. In terms of total citations (TC), China ranked first with 5889, and the United States followed closely with 5439. It is evident that China and the United States dominate both publications and citations in the fields of machine learning and liver cancer, positioning them at the forefront of research in this area. After these 2 countries, there is a significant drop in publication and citation volumes for other nations, indicating an imbalance in the global development of the field. Interestingly, 4 of the top 5 most-cited original articles and reviews come from the United States, showing that while the U.S. ranks second in publication volume, the quality and impact of its research are highly authoritative within the field.

**Table 3:** The top 10 impact countries/regions and institutions involved in LC.

Rank	Countries	Np	TC	AC	Institutions	Np
1	China	537	5889	10.97	Sun Yat-Sen Univ	48
2	USA	210	5439	25.90	Fudan Univ	37
3	Italy	45	656	14.58	Zhejiang Univ	32
4	Germany	44	988	22.45	Capital Med Univ	27
5	Canada	36	602	16.72	Chinese Acad Sci	26
6	South Korea	36	280	7.78	Southern Med Univ	26
7	India	34	342	10.06	Nanjing Med Univ	25
8	Japan	34	454	13.35	Zhengzhou Univ	25
9	France	31	802	25.87	Wenzhou Med Univ	24
10	England	29	575	19.83	Sichuan Univ	18

AC = average citations, LC/ML = liver cancer and machine learning, Np = number of papers, TC = total citations.

### 3.4. Top 15 most cited papers within the local citation range in LC studies

Highly cited publications are often viewed as significant contributions to a discipline, demonstrating substantial academic merit and influence. They serve as key indicators in bibliometric analyses. Tables 4 and 5 list the 15 most cited original research papers and reviews within the local citation scope of global liver cancer studies. The 15 most cited original research papers were predominantly published between 2001 and 2019, with an average impact factor of 51.80. The most cited paper is EASL Clinical Practice Guidelines: Management of Hepatocellular Carcinoma, which has been cited 99 times. Following closely is Diagnosis, Staging, and Management of Hepatocellular Carcinoma: 2018 Practice Guidance by the American Association for the Study of Liver Diseases, with 80 citations, and Radiomics: Images Are More than Pictures, They Are Data, with 78 citations. Notably, 3 of the top 4 papers are clinical guideline articles, highlighting the authority and accuracy of guidelines, which tend to attract considerable attention from researchers. Table 5 presents the 15 most cited review papers. The 2 most cited reviews were conducted by the American Cancer Society on cancer statistics, recognized for their authority and accuracy, which explains their high citation counts.

**Table 4:** The top 15 local cited original research related to the LC.

No.	Title	DOI	Year	First author	Citation
1	EASL clinical practice guidelines: management of hepatocellular carcinoma	10.1016/J.JHEP.2018.03.019	2018	European Association for the Study of the Liver	99
2	Diagnosis, staging, and management of hepatocellular carcinoma: 2018 practice guidance by the American Association for the Study of Liver Diseases	10.1002/HEP.29913	2018	Marrero, Jorge A	80
3	Radiomics: images are more than pictures, they are data	10.1148/RADIOL.2015151169	2016	Robert J. Gillies	78
4	AASLD guidelines for the treatment of hepatocellular carcinoma	10.1002/HEP.29086	2018	Heimbach, Julie K	56
5	Computational radiomics system to decode the radiographic phenotype	10.1158/0008-5472.CAN-17-0339	2017	Joost J.M. van Griethuysen	50
6	Radiomic analysis of contrast-enhanced CT predicts microvascular invasion and outcome in hepatocellular carcinoma	J.JHEP.2019.02.023	2019	Xun Xu	50
7	GSVA: gene set variation analysis for microarray and RNA-Seq data	10.1186/1471-2105-14-7	2013	Sonja Hänzelmann	46
8	Radiomics: Extracting more information from medical images using advanced feature analysis	10.1016/J.EJCA.2011.11.036	2012	Philippe Lambin	46
9	LIMMA powers differential expression analyses for RNA-sequencing and microarray studies	10.1093/NAR/GKV007	2015	Matthew E. Ritchie	44
10	Random forests	10.1023/A:1010933404324	2001	Breiman L	42
11	Deep learning with convolutional neural network for differentiation of liver masses at dynamic contrast-enhanced CT: a preliminary study	10.1148/RADIOL.2017170706	2017	Koichiro Yasaka	39
12	Radiomics machine-learning signature for diagnosis of hepatocellular carcinoma in cirrhotic patients with indeterminate liver nodules	10.1007/S00330-019-06347-W	2018	Fatima-Zohra Mokrane	37
13	Robust enumeration of cell subsets from tissue expression profiles	10.1038/NMETH.3337, 10.1038/NMETH.3337	2013	Aaron M Newman	37
14	clusterProfiler: an R Package for comparing biological themes among gene clusters	10.1089/OMI.2011.0118	2012	Guangchuang Yu	37
15	Predicting the grade of hepatocellular carcinoma based on non-contrast-enhanced MRI radiomics signature	10.1007/S00330-018-5787-2	2019	Minghui Wu	36

LC = liver cancer.

**Table 5:** The top 15 local cited reviews related to the LC.

No.	Title	DOI	Year	First author	Citation
1	Global cancer statistics 2018: GLOBOCAN estimates of incidence and mortality worldwide for 36 cancers in 185 countries	10.3322/CAAC.20107	2011	A. Jemal	132
2	Global cancer statistics 2020: GLOBOCAN estimates of incidence and mortality worldwide for 36 cancers in 185 countries	10.3322/CAAC.21660	2021	S. Hyuna	117
3	Radiomics: the bridge between medical imaging and personalized medicine	10.1038/NRCLINONC.2017.141	2017	Philippe Lambin	81
4	Hepatocellular carcinoma	10.1038/S41572-020-00240-3	2021	Josep M. Llovet	66
5	Hepatocellular carcinoma	10.1056/NEJMRA1713263	2019	Augusto Villanueva	61
6	A global view of hepatocellular carcinoma: trends, risk, prevention and management	10.1038/S41575-019-0186-Y	2019	Ju Dong Yang	55
7	Hepatocellular carcinoma	10.1016/S0140-6736(18)30010-2	2018	Alejandro Forner	47
8	BCLC strategy for prognosis prediction and treatment recommendation: the 2022 update	10.1016/J.JHEP.2021.11.018	2021	Maria Reig	37
9	Global cancer statistics 2020: GLOBOCAN estimates of incidence and mortality worldwide for 36 cancers in 185 countries	10.3322/CAAC.21660, 10.3322/CAAC.21660	2021	Hyuna Sung	34
10	Cancer statistics, 2021	10.3322/CAAC.21654	2021	Rebecca L. Siegel	30
11	Hepatocellular carcinoma	10.1016/S0140-6736(22)01200-4	2022	Prof Arndt Vogel	30
12	Management of hepatocellular carcinoma: an update	10.1002/HEP.24199	2011	Jordi Bruix	28
13	Immunotherapies for hepatocellular carcinoma	10.1038/S41571-021-00573-2	2021	Josep M. Llovet	26
14	Hepatocellular carcinoma	10.1016/S0140-6736(03)14964-1	2003	Josep M. Llovet	23
15	Hepatocellular carcinoma: epidemiology and molecular carcinogenesis	10.1053/j.gastro.2007.04.061	2007	Hashem B. El–Serag	22

LC = liver cancer.

### 3.5. Co-authorship analysis

#### 3.5.1. Co-authorship analysis of outstanding authors

**Figure 4.**
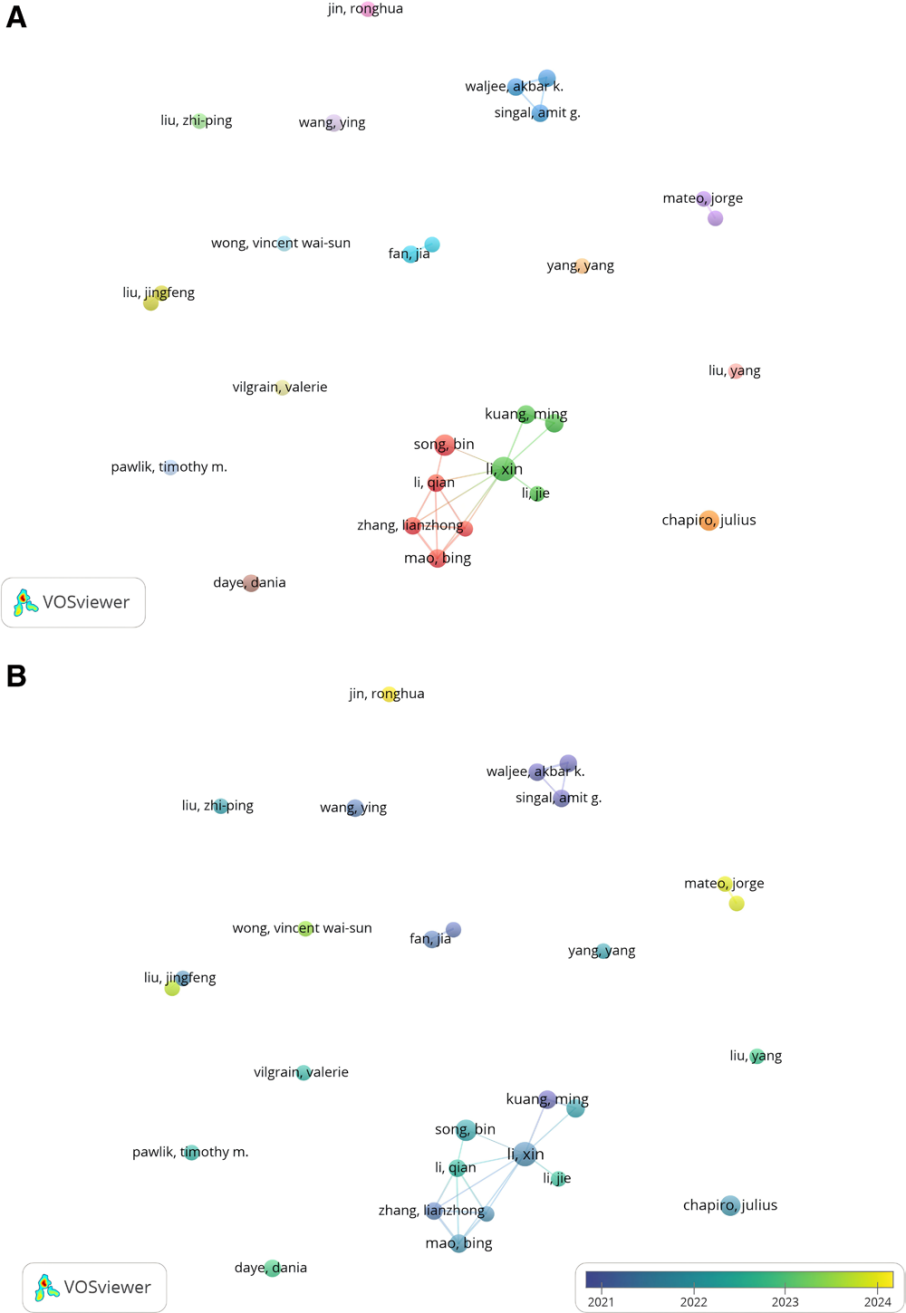
Co-authorship analysis of the influential authors in the field of LC/ML. (A) Network visualization map of collaborations among authors. (B) Overlay visualization map of collaborations among authors. LC/ML = liver cancer and machine learning.

Figure 4A presents a network visualization of the relationships among 28 coauthors, with a minimum publication threshold of 5. The map highlights authors such as “Li, Xin,” “Mao, Bing,” and “Zhang, Lianzhong,” who exhibit a higher number of collaborations and stronger connections within the network. While most authors are widely distributed across the network, there are relatively few instances of collaborative work. Authors like “Yang, Yang,” “Vilgrain, Valerie,” and “Pawlik, Timothy M.” primarily focus on individual contributions, suggesting a lack of a cohesive scholarly community in this field.

Figure 4B displays a temporal overlay of co-authorship patterns, tracking collaborative efforts over time. The analysis reveals that the landscape of contributing authors has evolved, with “Jing, Ronghua” and “Mateo, Jorge” emerging as key contributors in recent years, reflecting their growing influence in the field.

#### 3.5.2. Co-authorship analysis of the influential countries

**Figure 5.**
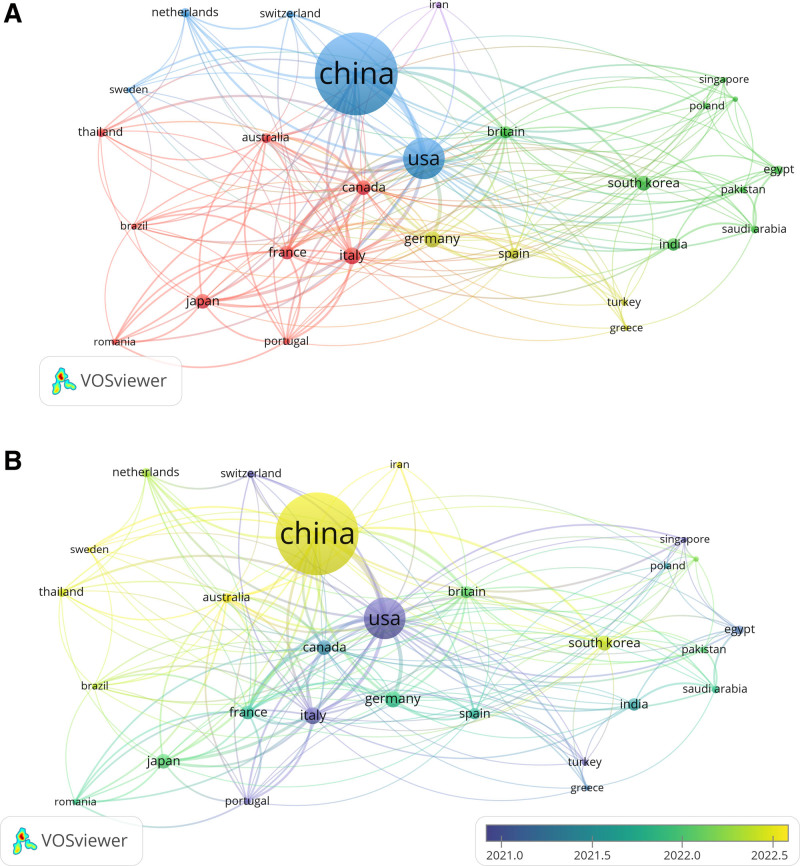
Co-authorship analysis of the influential countries (regions) in the field of LC/ML. (A) Network visualization map of collaborations among countries (regions). (B) Overlay visualization map of collaborations among countries (regions). LC/ML = liver cancer and machine learning.

Figure 5A illustrates the collaboration network of 31 countries, each with a minimum of 5 published documents. In this visualization, the size of each node represents the number of coauthored papers. China has the largest node, indicating the highest volume of coauthored publications, followed by the United States. While China leads in publication volume, the United States has established the most international collaborations. The figure displays several clusters, each represented by a distinct color. These clusters correspond to different international research groups. Cluster 1 (blue) is centered around China, Cluster 2 (red) around Italy, Cluster 3 (yellow) around Germany, and Cluster 4 (green) around South Korea. The lines connecting the nodes indicate the level of collaboration between countries (regions), with a particularly strong link between China and the United States. Our findings show that China is the most productive and engaged country in terms of collaborative research efforts. Figure 5B, a timeline-based visual representation, highlights a rise in international collaboration in recent years, a positive trend that promotes more balanced regional development. Increased cross-country collaboration not only advances knowledge but also drives practical innovation, benefiting all involved nations. Up until 2021, the United States was the central hub for international collaboration. However, by 2022, China had overtaken the US as the leading center for global research cooperation, driven in part by the significant prevalence of liver cancer in China.

#### 3.5.3. Co-authorship analysis of the active institutions

**Figure 6.**
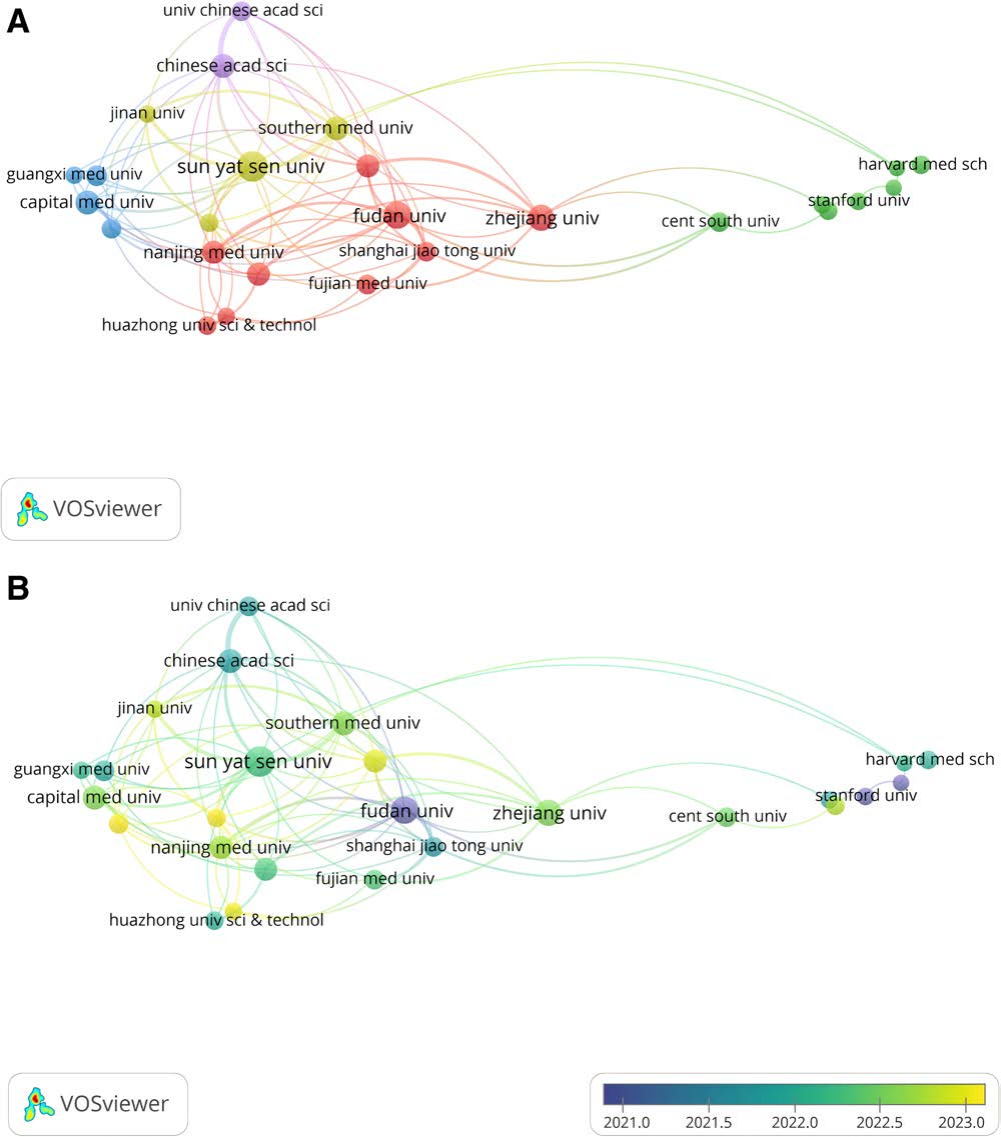
Co-authorship analysis of the influential institutions in the field of LC/ML. (A) Network visualization map of collaborations among institutions. (B) Overlay visualization map of collaborations among institutions. LC/ML = liver cancer and machine learning.

Figure 6A presents a collaborative network visualization for institutions with a minimum of 10 published articles. The analysis of institutional cooperation reveals that Sun Yat-sen University demonstrates a high level of collaboration, positioning it as one of the leading institutions in the field. Fudan University follows closely, contributing significantly to a strong collaborative team. Figure 6B illustrates the temporal distribution of institutional contributions, highlighting that Fudan University and Stanford University played pivotal roles in research efforts up until 2021. In conclusion, fostering innovation and progress in global LC/ML research will benefit from the proactive institution of academic conferences to promote further collaboration and development.

### 3.6. Co-citation analysis

#### 3.6.1. Co-citation analysis of cited-reference

**Figure 7.**
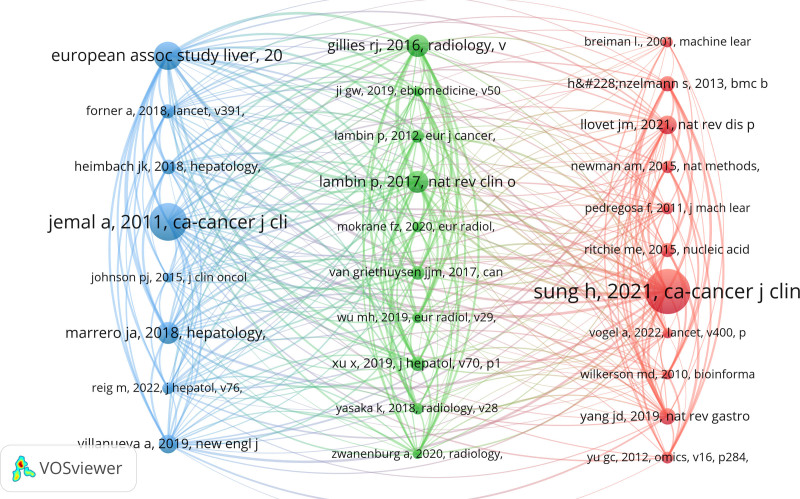
The visualization analysis of co-cited references in the field of LC/ML. LC/ML = liver cancer and machine learning.

Cocitations play a dual role in highlighting both the foundational literature that has significantly contributed to the development of a field and the current research trends. In the context of liver cancer and machine learning, co-citation patterns reveal the key studies that have shaped the discipline and provide insights into evolving research priorities. The network visualization map in Figure 7 shows 29 references with more than 34 cocitations, illustrating the central role LC/ML techniques have played in recent research. This demonstrates a strong scientific focus on applying machine learning to liver cancer, with several researchers emphasizing its potential. These co-cited references serve as both a foundation for ongoing work and an indicator of shifting research interests within the field.

#### 3.6.2. Co-citation analysis of cited sources (journal)

**Figure 8.**
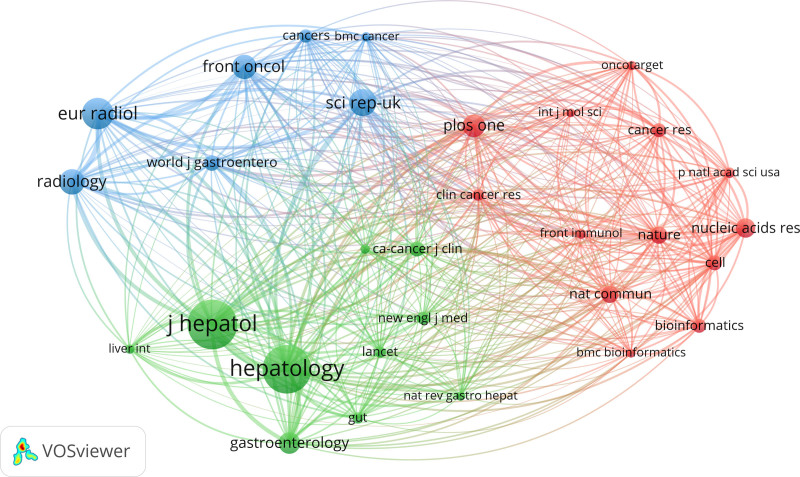
The visualization analysis of co-cited journals in the field of LC/ML. LC/ML = liver cancer and machine learning.

The co-citation network of journals, as illustrated in Figure 8, is divided into 3 distinct clusters, each represented by different colors. The green cluster includes journals from the field of hepatology, with a focus on gastrointestinal and liver diseases. The blue cluster corresponds to radiology journals, primarily addressing advancements in medical imaging and radiological studies. The red cluster is related to bioinformatics, emphasizing research at the molecular level and the application of bioinformatic technologies in liver cancer. These journals have contributed significantly to the field by publishing numerous key advancements in clinical cancer research, as well as comprehensive reviews on cancer prevention, diagnosis, and treatment.

### 3.7. Analysis of references burst

**Figure 9.**
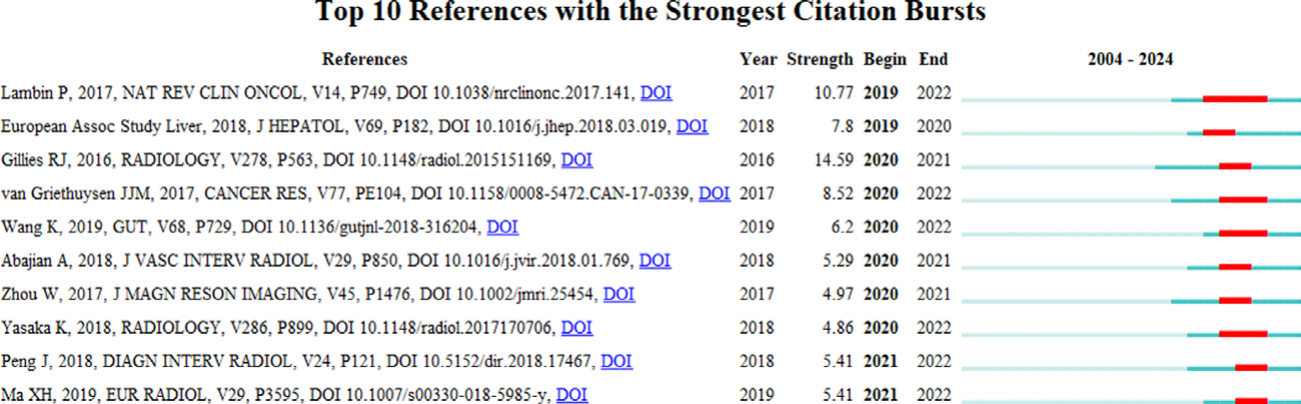
The visualization analysis of References Burst.

References with citation bursts are those that are frequently cited by scholars in a field over a period of time. In our study, CiteSpace identified and analyzed 10 references with strong citation bursts. Figure 9 shows that these bursts began as early as 2019 and continued until 2022. Each row in the table corresponds to a reference, and the red bars indicate periods of intense citation activity. The reference with the strongest citation burst (strength = 14.59) was authored by Gillies RJ, published in 2016, and appeared in the journal Radiology. This reference experienced a burst from 2018 to 2021, indicating that it gained significant recognition and citations during these years. Following closely, the reference with the second strongest citation burst (strength = 10.77) was authored by Lambin P. published in 2017 in Nature Reviews Clinical Oncology, which exhibited its citation burst from 2019 to 2022.

Overall, the burst strength for these references ranges from 4.86 to 14.59, indicating varying levels of influence within their respective burst periods. The endurance of these bursts also varied, typically ranging from 2 to 3 years, as evidenced by the time intervals displayed in the table. This distribution highlights the lasting impact and scholarly attention these references received during the identified periods, marking them as pivotal contributions to their fields.

### 3.8. Co-occurrence analysis of subject categories

**Figure 10.**
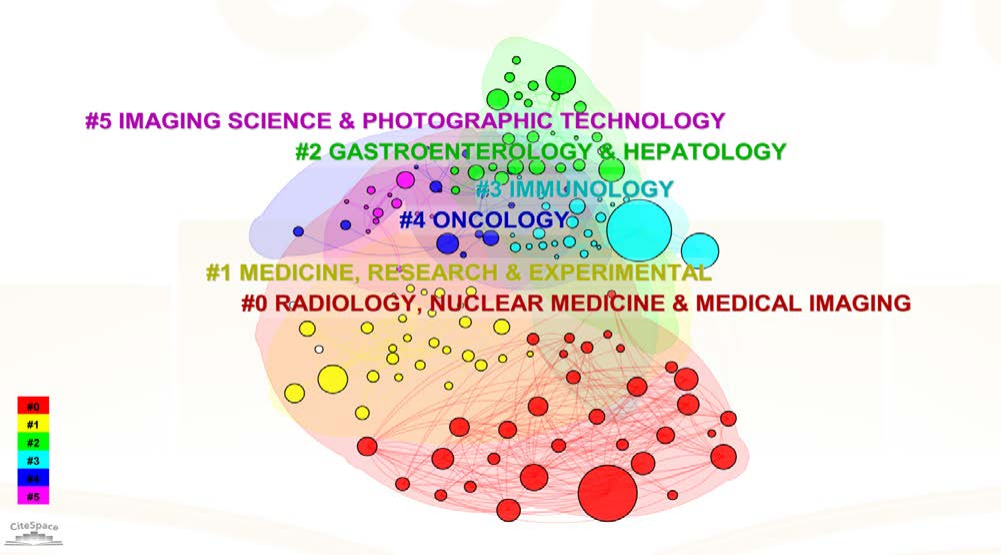
The visualization analysis of the co-occurrence of subject categories.

This section analyzes the co-occurrence of subject categories, illustrated in Figure 10, revealing 6 main clusters that represent key thematic areas within the research field. The core clusters include Radiology, Nuclear Medicine & Medical Imaging (#0), and Medicine, Research & Experimental (#1). Radiology and imaging form the largest and most central cluster, highlighting their broad interdisciplinary connections. The Medicine, Research & Experimental cluster, closely linked to radiology, underscores the foundational role of experimental research across various medical studies.

The specialized clusters encompass Gastroenterology & Hepatology (#2), Immunology (#3), and Oncology (#4), representing specific medical specialties that frequently intersect with the core areas of radiology and experimental medicine. These intersections suggest focused research applications, particularly in disease diagnosis and treatment. Additionally, the Imaging Science & Photographic Technology cluster (#5) is a smaller, specialized group, likely pointing to advancements in imaging technologies that support broader medical applications.

In summary, this co-occurrence analysis demonstrates that radiology and imaging are central to interdisciplinary research, with substantial connections to experimental medicine and specialized fields, especially in diagnostics and therapeutic developments.

### 3.9. Analysis of keywords

**Figure 11.**
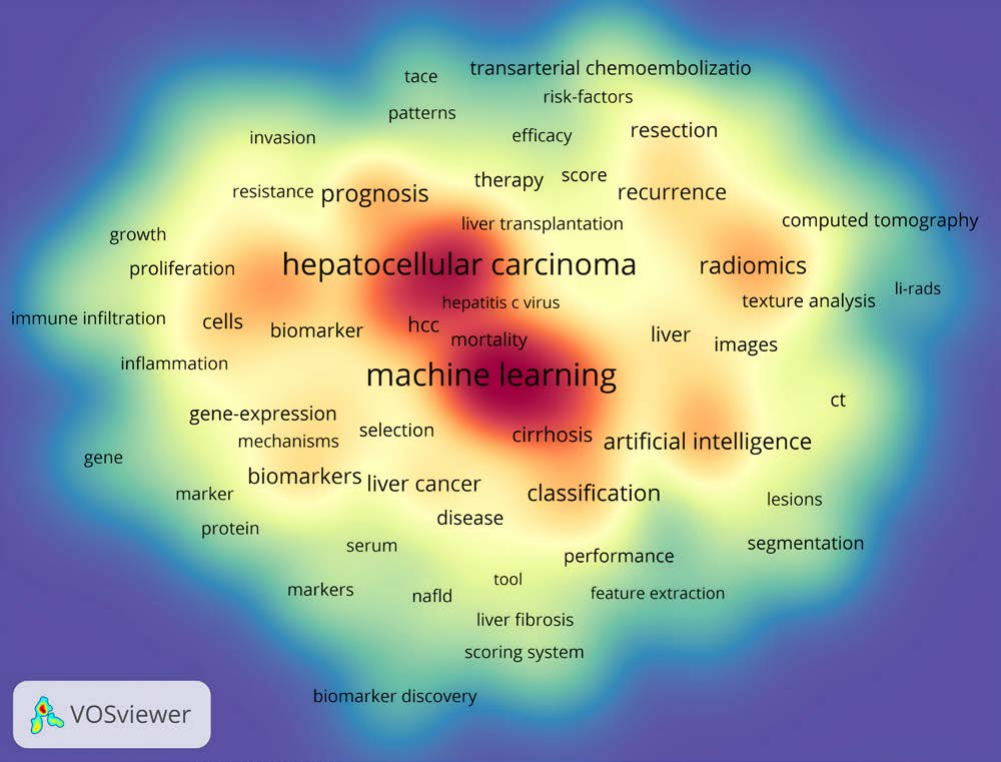
The visualization analysis of co-occurrence keywords in the field of LC/ML. LC/ML = liver cancer and machine learning.

The visual representation generated by Vosviewer illustrates the co-occurrence of keywords in this research field (Fig. 11). The most prominent keywords include “machine learning,” “hepatocellular carcinoma,” “cancer,” and “radiomics,” which highlight the core areas of study within LC/ML. Table 6 presents the top 20 keywords in frequency of occurrence. These terms represent significant aspects of the research focus.

**Table 6:** Top 20 keywords in frequency of occurrence.

No.	Keyword	Frequency	TLS
1	Machine learning	434	2591
2	Hepatocellular carcinoma	355	2096
3	Hepatocellular-carcinoma	210	1250
4	Cancer	188	1105
5	Diagnosis	123	861
6	Prognosis	119	762
7	Radiomics	118	900
8	Expression	109	651
9	Artificial intelligence	81	643
10	Survival	81	516
11	Prediction	77	587
12	Classification	76	503
13	Deep learning	75	601
14	Recurrence	69	540
15	Risk	67	428
16	Liver cancer	62	356
17	Cells	57	309
18	Resection	56	432
19	Biomarkers	55	338
20	Liver	53	362

TLS = total link strength.

**Figure 12.**
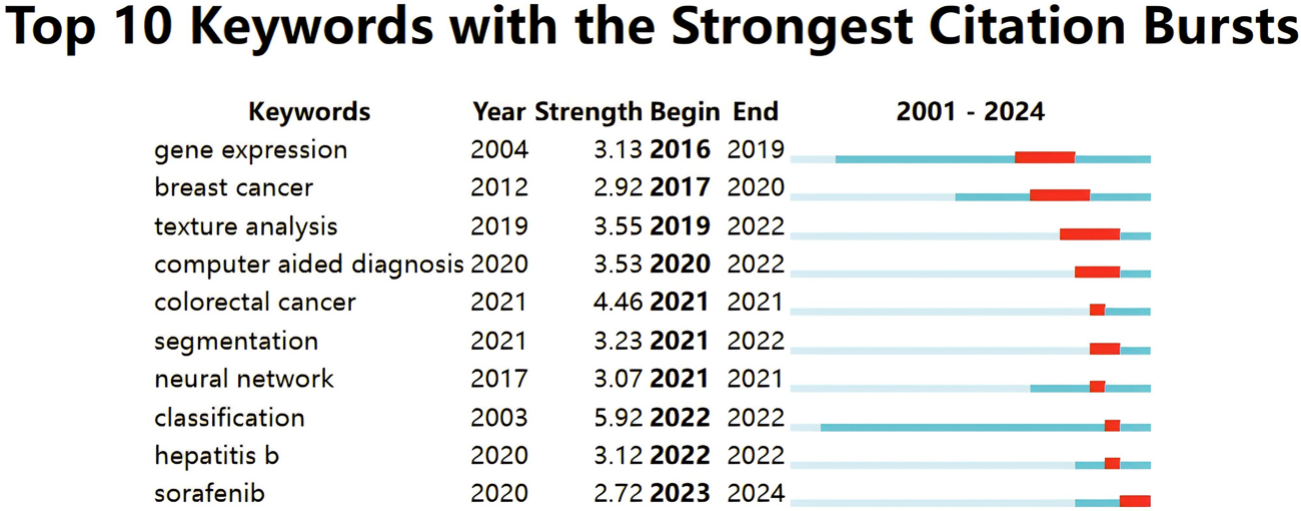
The top 10 keywords with the strongest citation bursts in the field of LC/ML. LC/ML = liver cancer and machine learning.

To better understand the recent surge in interest in LC/ML, CiteSpace’s Burst analysis feature provides valuable insights into evolving trends. Figure 12 shows the top 10 terms with the most pronounced citation bursts. Earlier research hotspots, such as “gene expression,” “breast cancer,” and “texture analysis,” suggest that past studies focused primarily on the pathogenesis of LC and the integration of machine learning. More recent keywords, including “computer-aided diagnosis,” “segmentation,” and “classification,” indicate a shift toward the application of machine learning in liver cancer, with greater emphasis on diagnosis and treatment.

## 4. Discussion

The global public health sector continues to face significant challenges in cancer prevention and treatment, with liver cancer being a focal point of concern. Despite growing attention, several unresolved issues remain, such as the lack of LC-specific molecular research, resistance to multiple chemotherapeutic drugs, and the limited success of surgical interventions. In recent years, there has been increasing interest in applying machine learning within artificial intelligence to improve the diagnosis and treatment of LC. This has led to expanded research across various areas, including screening, diagnosis, grading, subtype classification, disease assessment, management, and treatment of LC. This study conducted a bibliometric analysis to explore the connection between ML and the clinical diagnosis and treatment of LC. Our goal was to provide researchers with a comprehensive overview of the current state of ML applications in LC and to highlight the future potential of these technologies in improving diagnosis and treatment outcomes.

### 4.1. Analysis of characteristics of literature

The number and trajectory of publications in a scientific field often reflect the maturity of that discipline. Research on liver cancer was in its early stages between 2001 and 2012, with fewer than 10 articles published annually. From 2013 to 2015, the field experienced moderate growth, followed by rapid expansion from 2016 to 2024, peaking at 269 publications in 2023 before a slight decline in 2018. Early progress in LC detection and treatment was driven by innovations such as alpha-fetoprotein (AFP) antibody-coupled fluorescent magnetic probes in 2003.^[29]^ A major turning point occurred in 2006 with the introduction of recursive support vector machine methods for gene and biomarker prioritization. By 2011, metabolomics studies using ultra-high-performance liquid chromatography combined with electrospray ionization and quadrupole flight time mass spectrometry provided new insights into hepatocellular carcinoma pathophysiology.^[31]^ The subsequent growth in related research can be attributed to key findings in 2015 and 2017,^[32]^ which highlighted the role of DNA methylation and long non-coding RNAs in hepatocellular carcinoma development.

In terms of journal impact, Nature Medicine recorded the highest total citations at 706, followed by European Radiology (TC = 565) and Scientific Reports (TC = 524). Frontiers in Oncology ranked first in H-Index (13), with Scientific Reports second (H-Index = 12), and Cancers and World Journal of Gastroenterology tied for third (H-Index = 9). Journals with high TC and H-Index are widely read by experts and academics, signaling greater recognition and influence of the research findings.

While factors such as country, institution, or authorship do not directly determine the quality of a paper, bibliometric data reveal its impact within a specific field. Most of the publications in LC/ML come from the People’s Republic of China, followed by the United States, Italy, Germany, and Canada. China has the highest number of publications, and combined with the US, these 2 countries account for over 50% of the total output, demonstrating their leadership in this field. This dominance likely stems from strong commitments to ML in LC clinical research, supported by public and private sector funding and similar policies in both nations. A 2020 study found that China and the US are leading in cancer cell treatment research, with China conducting more clinical trials. However, while China produces a large volume of research, its average citation count is lower than that of other countries (regions), and it has fewer highly cited papers, indicating room for improvement in research quality.^[32]^ Nonetheless, China’s strong research capabilities are evident, as 4 of the top ten institutions contributing to LC/ML papers are based in China. Sun Yat-sen University, in particular, has built an excellent international reputation due to its substantial research output and groundbreaking discoveries. Among the top 10 authors in the field, 8 are from China, highlighting the significant role Chinese scholars play in advancing LC/ML research.

### 4.2. Historical evolution of LC/ML research

The evolution of liver cancer and machine learning research can be traced through citation networks and highly cited publications. Between 2001 and 2012, few academic papers were published on this topic. One early example is a 2003 study on the clinical use of surgical robotics in treating hepatocellular carcinoma.^[33]^ In 2009, a global review highlighted laparoscopic liver surgery as a safe and effective method for managing liver diseases. However, the study emphasized the need for hepatobiliary and laparoscopic surgeons to develop specialized skills. The widespread adoption of laparoscopic liver surgery has expanded the range of treatments available, advancing surgical techniques.^[34]^ From 2013 to 2015, LC/ML research began to increase gradually. A 2014 study compared robotic and laparoscopic surgeries in hepatic resection, concluding that while robotic surgery involved more fully minimally invasive procedures, it did not show significant advantages in surgical outcomes over laparoscopic techniques. This study provided important insights for surgical management in liver cancer.^[35]^ Between 2016 and 2024, research into ML applications in LC diagnosis and treatment grew steadily. A 2016 study explored the potential of imaging genomics (radiogenomics) in improving clinical decision-making for cancer patients, specifically in liver cancer diagnosis, treatment, and prognosis. The study underscored the importance of integrating imaging genomic data for advancing cancer therapies.^[36]^ In 2018, a study demonstrated that combining baseline MR imaging with artificial intelligence and ML techniques could predict outcomes of transarterial chemoembolization in hepatocellular carcinoma patients.^[37]^ Similarly, a 2019 study used contrast-enhanced computed tomography and large-scale clinical data to investigate the prognosis of HCC patients following radical surgical resection, further highlighting the significant role of ML in liver cancer management.^[38]^

It is also noteworthy that clinical trials related to LC/ML (ChiCTR2300072677, NCT05543304, ChiCTR2200061765, ChiCTR200003696, NCT03198975) have predominantly been conducted in China, as shown by the International Clinical Trials Registry Platform link. In conclusion, research on LC/ML has grown significantly in recent years, and future studies in this area continue to show promising potential.

### 4.3. Research priorities and future directions for ML applications to LCs

Our analysis of cocitations and keyword co-occurrences within the LC and ML research fields suggests that future research will primarily focus on clinical applications related to the diagnosis and treatment of hepatocellular carcinoma. These emerging priorities indicate a shift toward utilizing machine learning to enhance diagnostic accuracy, improve treatment outcomes, and support personalized care strategies for liver cancer patients.

#### 4.3.1. Diagnosis

Timely identification of liver cancer is crucial in reducing mortality rates associated with the disease. Traditionally, diagnosis begins with an assessment of a patient’s clinical symptoms, which guides further investigations, including physiological tests and imaging techniques such as computed tomography (CT) scans to identify lesions. Pathological examination of tissue samples is also used to confirm the presence of cancer and determine its severity. Machine learning is increasingly being integrated into this diagnostic process. By leveraging high-performance computing and patient data, ML techniques can quantify and analyze a patient’s full medical history, including clinical symptoms and subjective complaints, to predict the progression of liver cancer. This approach has been shown to reduce misdiagnoses and the overuse of medications. Early-stage patients exhibiting abnormal clinical symptoms can benefit from laboratory tests, where blood and urine samples are analyzed for markers such as AFP and γ-glutamyl transpeptidase, helping to identify liver cancer in its initial phases. In the coming years, ML models will continue to evolve, utilizing large datasets to enhance diagnostic accuracy. These models will focus on gene enrichment analysis, identifying key biomarkers and molecular targets for cancer treatment and immunotherapy. The ultimate goal is to improve disease prediction, reduce testing and diagnosis times, and optimize diagnostic methodologies.^[39]^ Low-dose CT scans have become the primary screening tool for high-risk individuals, and computer-aided diagnosis systems are being developed to assist clinicians in interpreting medical imaging data more accurately.^[40]^ By comparing ML datasets, abnormalities in CT images can be detected, and tissue samples from suspicious lesions can be further analyzed through pathology. Pathological images of tissue samples play a vital role in early cancer detection, disease monitoring, and prognosis, while also helping to guide treatment strategies and improve therapeutic outcomes. However, the growing population in China and the rapid accumulation of imaging data have placed significant pressure on clinical physicians. To alleviate this burden, it is crucial to improve ML-driven data processing and image analysis systems. Automated medical image processing systems are essential for increasing efficiency, reducing medical errors, and improving hospital workflows, aiming for faster, deeper, and more accurate diagnostic processes.

#### 4.3.2. Main treatment methods

Hepatocellular carcinoma is a major global health concern, ranking as the third leading cause of cancer-related deaths, with approximately 700,000 fatalities annually.^[41]^ At present, there exist 2 distinct categories of therapeutic options for LC, namely those amenable to surgical resection and those presenting challenges for complete eradication by surgical means. The most effective course of treatment for liver cancer is contingent upon the specific stage and kind of cancer. Available treatment options include localized interventions such as surgery, ablation, embolization, and radiation, as well as systemic therapies including targeted therapies, immunotherapies, and chemotherapies. The advancement of artificial intelligence has led to enhancements in machine learning techniques for improving the survival rates of patients with liver cancer. This is achieved through the integration and analysis of extensive and complex datasets that comprehensively describe clinical treatments for liver cancer. By utilizing various perspectives derived from this accumulated data, survival assessments can be conducted.

In order to determine the potential risk of surgery for promising surgically resectable LC, a thorough preoperative assessment is necessary. This assessment involves employing machine learning techniques, wherein textural features of LC tumor lesions in CT images are measured and combined with the patient’s clinical variables as input features. These input features are then used to train ML classifiers, such as logistic regression or linear discriminant analysis, to estimate the risk of malignant tumor development. In general, the measures including the diameter size, spatial coordinates, quantity, and demarcations of the malignant lesions seen in the CT scans are used to aid in evaluating the appropriateness of surgical resection as a therapeutic option. Nevertheless, in the event that the preoperative evaluation indicates that the patient guarantees an adequate residual liver functional reserve for compensation, it is recommended to perform a surgical resection of the lesion that ensures a minimum distance of 1 cm between the surgical margins and the tumor border. Alternatively, the surgical margins should be thoroughly examined to confirm their absence of tumor presence. This approach is considered crucial for attaining long-term survival outcomes in patients with LC. The utilization of imaging data, particularly CT and MRI, in conjunction with ML during the postoperative phase is crucial for verifying the complete eradication of residual tumors. In cases where preoperative AFP levels are elevated, it is imperative to conduct quantitative AFP testing 2 months after the surgery to ascertain that AFP levels have decreased to within the normal range. Nevertheless, in the event that the preoperative assessment indicates a total absence of liver function, liver transplantation emerges as a remedial intervention for individuals afflicted with liver cirrhosis. Due to the scarcity of liver resources, the process of liver transplantation necessitates doctors to exercise meticulousness in the selection of a suitable liver, taking into consideration many parameters such as tumor stage, AFP level, and cumulative dose of immunosuppressants. ML techniques are employed to develop robust predictive models in the field of transplant medicine. These models utilize data to assess the accuracy of transplant pairing, evaluate the effectiveness of graft allocation, and predict various outcomes such as patient survival, graft rejection/failure, and postoperative morbidity. By providing more precise prognoses, diagnoses, and identification of risk factors, these predictive models facilitate expedited decision-making during the graft allocation process. Consequently, the time required to match liver transplant recipients with suitable livers is reduced. The conventional method of postoperative prognosis is dependent on the expertise and experience of physicians, who rely on previous patient history and medical data. Nevertheless, research has shown that doctors often exhibit subpar performance when it comes to prognostication and estimating anticipated survival rates, frequently demonstrating a tendency to overestimate the duration of life.^[42–44]^ On the other hand, ML has demonstrated promise in various datasets such as genomic, transcriptomic, proteomic, and radiomic data for the purpose of predicting patient prognosis and survival. Furthermore, the incorporation of ML in surgical treatment has the potential to be effectively integrated into the routine practice of physicians, aiding in their examination of disease progression and therapeutic considerations for patients with liver cancer. Insufficient remuneration of remaining hepatic function stands as a primary factor impeding the comprehensive excision of liver cancer. In the case of these patients, a viable alternative is to consider preoperative hepatic artery chemoembolization or external radiation treatment as a means to promote tumor shrinking, followed by subsequent surgical resection.^[45,46]^ Transarterial chemoembolization is a frequently used non-surgical approach utilized in the treatment of LC.^[47–50]^ Distinguishing between reactive and non-reactive nodules or new lesions on CT scans during therapy poses a significant challenge for radiologists. However, the reliable assessment of this differentiation is currently accomplished via the use of machine learning algorithms that analyze large datasets. These algorithms play a crucial role in determining the suitability of surgery or the need for second-line treatment.^[51]^ Chemotherapeutic drug particle emboli include a range of materials, such as conventional gelatin sponge particles, polyvinyl alcohol particles, microspheres, and drug-eluting beads. Drug-eluting beads, in particular, represent a unique embolization agent that facilitates the delivery of chemotherapeutic drugs. The use of drug-eluting beads transarterial chemoembolization (DEB-TACE) has been on the rise among patients diagnosed with numerous solid malignancies.^[52,53]^ In order to assure the safety of using DEB-TACE, a prediction tool produced by ML was used to forecast the likelihood of acute liver function deterioration subsequent to DEB-TACE. The integration of clinical and biological aspects has shown to be effective in clinical practice, thanks to the progress made in machine learning algorithms.^[54,55]^ Hence, the use of a unique machine learning-based prediction tool enables physicians to adapt treatment regimens in order to enhance individualized care. This is achieved by the analysis of preoperative clinical and biological parameters of patients with hepatocellular carcinoma who are receiving DEB-TACE. Radiation therapy serves as a palliative intervention for LC, effectively eliminating malignant cells through the application of radiation. This treatment modality encompasses both external radiation therapy, where radiation is administered from outside the body using an external beam, and internal radiation therapy, which involves the implantation of radiation particles. Examples of internal radiation therapy techniques include Y-90 microsphere therapy, iodine-131 monoclonal antibody,^[56]^ and radioactive iodine oil. Nevertheless, adjacent normal tissues and essential organs in the vicinity of the target region may also experience adverse effects, resulting in radiotherapy-induced toxicity and harm to healthy bodily tissues. Hence, in the process of devising a radiation treatment regimen, it is essential to carefully consider the prospective advantages in relation to the possible harm inflicted upon healthy organs and tissues. The primary objective is to optimize the therapeutic outcome while simultaneously reducing the probability of adverse effects on normal tissue. Therefore, by the use of machine learning techniques to predict and categorize difficulties resulting from radiation, this study aims to address both methodological and clinical considerations. By accurately predicting and assessing the toxicity of different radiotherapy regimens, it is possible to mitigate the side effects associated with this treatment modality.

## 5. Conclusion

Liver cancer research has a long and rich history, tracing back to the late 19th century. Over the past century, advancements in social economics, science, and technology have driven significant progress in the field. Machine learning, as a key component of artificial intelligence, has played an increasingly important role, especially in clinical diagnosis, therapeutic strategies, and fundamental research. The application of ML in LC research continues to evolve, bringing constant advancements. This study analyzed the development of ML in the clinical aspects of LC over the past 2 decades, using tools like VOSviewer, CiteSpace, and CitNetExplorer. The objective was to systematically review the field’s progress, highlight influential authors, identify key institutions and journals, and analyze keyword clusters. Based on the findings, the following conclusions were drawn:

1. The formation of a collaborative network of researchers in the LC/ML field is well underway, with notable scholars participating, indicating a growing trend toward international collaboration.
2. Several prominent academic journals, such as Frontiers in Oncology, Scientific Reports, and Cancers, have emerged as key platforms for publishing LC/ML-related studies.
3. China and the United States are leading actors in global collaboration in this field. While Chinese scholars contribute significantly to publication volume, American-authored papers are more widely cited and recognized in the LC/ML domain.
4. Keyword clustering and co-occurrence analysis indicate a recent shift in LC/ML research towards clinical applications, offering stronger support for clinicians in diagnosing and treating liver cancer.

## 6. Limitations and outlook

Liver cancer, a significant disease with a long historical background, remains a persistent challenge in modern medicine, offering numerous opportunities for further advancement. The role of machine learning in LC research is vital, particularly in clinical diagnosis, therapy, prognosis, and fundamental research. This study represents the first systematic and comprehensive effort to map the characteristics and trends of literature related to the intersection of ML and LC in clinical contexts. It provides a framework for researchers to better understand the main areas of interest and emerging trends, while also offering guidance and inspiration for future investigations. In addition, this study identifies key journals and influential scholars in the LC/ML field, helping researchers efficiently locate relevant references for their work. It also provides direction for scholars seeking appropriate outlets for publication in this area. However, some limitations must be acknowledged. Although the Web of Science Core Collection database includes high-quality publications, some bibliographic omissions are inevitable. Additionally, recently published, high-quality articles may not yet have achieved the same citation impact as older, established works, leading to potential underrepresentation in the analysis.

## Author contributions

**Conceptualization:** Weimin Gao, Wang Liu.

**Data curation:** Yafen Wang, Yanchao Tang, Wanrong Wang, Lingyan Zhou, Yanjun Chen, Pengman Li, Bangjie Chen.

**Formal analysis:** Yafen Wang, Yanchao Tang, Wanrong Wang, Lingyan Zhou, Yanjun Chen, Pengman Li, Bangjie Chen.

**Project administration:** Weimin Gao, Wang Liu.

**Visualization:** Yafen Wang, Pengman Li, Bangjie Chen.

**Writing – original draft:** Enba Zhuo, Wenzhi Yang.

**Writing – review & editing:** Enba Zhuo, Yafen Wang, Yanchao Tang, Wanrong Wang, Lingyan Zhou, Yanjun Chen, Pengman Li, Bangjie Chen, Weimin Gao, Wang Liu.

## Supplementary Material


