# Supplementary material for: Global trends in machine learning applied to clinical research in liver cancer: Bibliometric and visualization analysis (2001–2024)
Source: Medicine (Baltimore). 2024 Dec 6;103(49):e40790. doi: 10.1097/MD.0000000000040790 (PMC11631000; doi:10.1097/MD.0000000000040790)
Supplement: Supplementary file 1 [file medi-103-e40790-s001.docx]

**Search strategy：**

TS= (“machine Learning” OR “machine study” OR “machine learn” OR “machines Learning” OR “machine learning” OR “robotic learning” OR “machine learning algorithm” OR “robot learning”) AND TS= (“liver cancer” OR “Cancer of the liver” OR “hepatocellular carcinoma” OR “hepatocellular cancer” OR“Malignant tumor of liver” OR “Hepatoma” OR“hepatocarcinoma” OR “liver carcinoma” OR “hepatic cancer” OR “hepatic carcinoma”)

**Query link:**

https://www.webofscience.com/wos/woscc/summary/d373c2f6-1123-4e11-8bd0-20a73e345fc2-4193b34e/times-cited-descending/1

(The results of this search may change slightly due to updates of the WoS database.)
